# How Trustworthy Are the Genomic Sequences of SARS-CoV-2 in GenBank?

**DOI:** 10.3390/microorganisms12112187

**Published:** 2024-10-30

**Authors:** Xuhua Xia

**Affiliations:** 1 Department of Biology, University of Ottawa, Marie-Curie Private, Ottawa, ON K1N 6N5, Canada; xxia@uottawa.ca; 2 Ottawa Institute of Systems Biology, University of Ottawa, Ottawa, ON K1H 8M5, Canada

**Keywords:** SARS-CoV-2, COVID-19, GenBank, data validation, genome, genomic analysis

## Abstract

Well-annotated gene and genomic sequences serve as a foundation for making inferences in molecular biology and evolution and can directly impact public health. The first SARS-CoV-2 genome was submitted to the GenBank database hosted by the U.S. National Center for Biotechnology Information and used to develop the two successful vaccines. Conserved protein domains are often chosen as targets for developing antiviral medicines or vaccines. Mutation and substitution patterns provide crucial information not only on functional motifs and genome/protein interactions but also for characterizing phylogenetic relationships among viral strains. These patterns, together with the collection time of viral samples, serve as the basis for addressing the question of when and where the host-switching event occurred. Unfortunately, viral genomic sequences submitted to GenBank undergo little quality control, and critical information in the annotation is frequently changed without being recorded. Researchers often have no choice but to hold blind faith in the authenticity of the sequences. There have been reports of incorrect genome annotation but no report that casts doubt on the genomic sequences themselves because it seems theoretically impossible to identify genomic sequences that may not be authentic. This paper takes an innovative approach to show that some SARS-CoV-2 genomes submitted to GenBank cannot possibly be authentic. Specifically, some SARS-CoV-2 genomic sequences deposited in GenBank with collection times in 2023 and 2024, isolated from saliva, nasopharyngeal, sewage, and stool, are identical to the reference genome of SARS-CoV-2 (NC_045512). The probability of such occurrence is effectively 0. I also compile SARS-CoV-2 genomes with changed sample collection times. One may be led astray in bioinformatic analysis without being aware of errors in sequences and sequence annotation.

## 1. Introduction

Molecular sequence databases hosted in NCBI/EMBL/DDBJ (U.S. National Center of Biotechnology Information, European Molecular Biology Laboratory, and DNA Data Bank of Japan) are the most important bioinformatic resources for modern biological and biomedical research worldwide. GenBank, as one of the databases, has been the most frequently used resource for functional and comparative genomics. Well-annotated gene and genomic sequences pave the way for a variety of inferences about gene functions as well as interactions among genes and their products. The first SARS-CoV-2 genome was submitted to GenBank [1] and immediately used to develop two successful COVID-19 vaccines [2,3]. The genomic resources also facilitated critical evaluation of the mRNA optimization in the development of the two vaccines [4] and a detailed understanding of the domain structure and function of the viral spike protein [5]. The many submitted SARS-CoV-2 genomes enabled many studies to date the most recent common ancestor (MRCA) of the sequenced SARS-CoV-2 genomes [6,7,8,9,10,11]. To facilitate this endeavor, NCBI staff have assembled very large phylogenies based on aligned SARS-CoV-2 genomes using NCBI’s C++ toolkit [12]. Such trees, with the collection time for each genome, have been used to date the common ancestors of the sampled SARS-CoV-2 genomes and to estimate their evolutionary rates, with unprecedented resolving power [10,11,13]. The aligned genomes also showed that SARS-CoV-2 exhibited extreme CpG deficiency, leading to the inference that the virus is under the selection of human zinc-finger antiviral proteins [14]. This inference was quickly substantiated by experimental evidence [15,16,17].

Almost all the inferences above require high-quality sequences, accurate annotations, and, above all, authentic data. Sequencing errors can often be reduced by improving the sample quality and increasing the sample size and sequencing depth/coverage [18,19,20,21]. Wrong annotations can often be detected and corrected. However, it is far more difficult to validate the authenticity of a genomic sequence. If one takes an existing sequence, makes a few random nucleotide replacements, and resubmits to GenBank as a new sequence, it is theoretically impossible to discriminate between this fake sequence and a real sequence.

In this paper, I take an innovative but admittedly low-power approach to detect sequences that cannot possibly be authentic. I also compile a partial list of SARS-CoV-2 genomes in GenBank with altered collection times, as well as some genomes that have been submitted but withdrawn. The results highlight the urgency of implementing quality control of sequence submission to GenBank [22].

### 1.1. Rationale for Identifying Inauthentic SARS-CoV-2 Genome Sequences in GenBank

I illustrate the rationale with the reference genome of SARS-CoV-2 (NC_045512), which was sampled at time *T* (26 December 2019), and an evolutionary rate of 0.05526/genome/day estimated from a phylogeny of 83,688 full-length and high-quality SARS-CoV-2 genomes [10]. The evolutionary rate, r, has also been estimated in several other studies [23,24,25,26] using other methods, with a clock changing linearly over time [11,13] or various uncorrelated relaxed clock models [27,28,29]. The estimated evolutionary rate in these studies is expressed as the number of changes per site per year and varies from low values, such as 0.0006 [23] and 0.000605 [24], to substantially higher values of 0.001793 [25] and 0.0024 [26]. Such variation is expected considering that the estimation involves factors that cannot always be controlled for [30]. One needs to multiply the rate above by a factor of (30,000/365) to obtain the number of changes per genome per day. The two slow rates would become 0.0493 and 0.0497/genome/day, and the two high rates would become 0.1474 and 0.1973/genome/day.

Suppose a genome, *S*, identical to NC_045512, was sampled at time *T + δ* (e.g., 18 January 2024, so δ=1484 days). Considering the evolutionary rate, *r* = 0.05526/genome/day, the expected number of nucleotide differences between the two genomes over the period of *δ* is as follows:

(1) λ=rδ=0.05526×1484=82.0058 

Assuming that mutations are random, we can use the Poisson distribution to find the probability of no nucleotide differences between genome *S* and the reference genome. This probability mass is as follows:

(2) $$f\left(0|\lambda \right)=e^{-\lambda }=2.4284\times 10^{-36}$$

This calculation shows that the probability of obtaining a SARS-CoV-2 genome, *S*, on 18 January 2024 that is identical to NC_045512 is effectively 0, even if billions of SARS-CoV-2 genomes were sequenced. This is true even when the slowest reported rate is used in the calculation. Such a genome, *S*, identical to NC_045512 but sampled on 18 January 2024, would be deemed inauthentic (as a mild form for fake). Note that the probability in Equation (1) could be even smaller for two reasons. First, a viral genome can change not only through point mutations but also through insertions and deletions (indels). The formulation of this probability in Equation (2) considered only point mutations. If indels also occur, then the chance of finding an exact copy of NC_045512 on 18 January 2024 would be even smaller. Second, the formulation in Equation (2) assumes that all SARS-CoV-2 strains are descendants of the reference genome NC_045512. If the subsequently dominant SARS-CoV-2 strains are not direct descendants of NC_045512, then the probability that we would obtain a genome at time *T + δ* that is identical to NC_045512 would be smaller.

### 1.2. Identifying SARS-CoV-2 Genomes in GenBank with Altered Collection Times

Changes made to the collection times of viral samples are not recorded in GenBank. If the originally reported collection time was subsequently modified, only the modified collection time will appear in the GenBank sequence file. This creates difficulties in identifying which SARS-CoV-2 genomes have a modified collection time.

Fortunately, NCBI has routinely compiled full-length high-quality SARS-CoV-2 genomes and built phylogenetic trees as a service to the public (https://www.ncbi.nlm.nih.gov/labs/virus/vssi/#/precomptree, accessed multiple times up to 7 May 2022). The OTU names in the tree include GenBank accession and collection times. One may download two trees at times T1 and T2. If a SARS-CoV-2 genome appears in both trees but with different collection times, then a modification of the collection time has occurred during the interval between the compilation of the two trees.

## 2. Materials and Methods

### 2.1. Identify Inauthentic Sequences

I downloaded early SARS-CoV-2 genomes and searched for identical sequences in GenBank. A stringent criterion of identity was used, i.e., two sequences were considered identical if they were exact copies of each other (identical in both sequence length and nucleotide sequence). For those identical genomes thus identified, I calculated the probability of their occurrence according to Equation (1) as the basis for judging the authenticity of these sequences based on the probability.

### 2.2. Identify Genomes with Altered Collection Times

NCBI released phylogenetic trees of SARS-CoV-2 genomes continuously. I downloaded seven trees on 3 April, 25 April, 29 May, 12 July, 4 September, and 8 November 2021, and 7 May 2022. These trees are hereafter referred to as Apr3_21, Apr25_21, May29_21, Jul12_21, Sept4_21, Nov8_21, and May7_22, respectively, and contain 86,582, 142,591, 183,347, 304,221, 459,944, 633,995, and 978,217 SARS-CoV-2 genomes, respectively. SARS-CoV-2 genomes in an early tree do not represent a subset of those in a late tree. For example, 4850 genomes in the Apr3_21 tree are absent in the Sep4_21 tree, 2412 genomes in the Sep4_21 tree are absent in the Nov8_21_tree, and 4473 genomes in the Nov8_21 tree are absent in the May7_22 tree. This is partly because some SARS-CoV-2 genomes submitted to GenBank were subsequently withdrawn by the submitters (e.g., FR988889, FR988892, FR988974, FR989034, and FR988093).

For SARS-CoV-2 genomes present in two trees, their collection times were compared. For example, the isolation time for genome MW750862 was recorded as 22 May 2020 in one tree but 2 March 2021 in a later tree. Such differences in the collection time between trees were recorded. This means that, when the genomic sequence was originally submitted, the collection time was recorded as 22 May 2020, but this collection time was subsequently changed to 2 March 2021 in the GenBank record. The GenBank file does not record the alteration of collection dates, and only the altered collection date, i.e., 30 December 2021, is visible to users accessing the GenBank record. For those SARS-CoV-2 genomes that are present in only one tree, it is now impossible to know whether their collection dates have been altered or not unless other information is available. For example, the SARS-CoV-2 genome from Utah, USA (MW795884), was present in the Nov8_21 tree but not in the May7_22 tree. Although the collection time of this sequence has been changed from 13 January 2020 to 13 January 2021, the change will not be detected by the described comparisons between the two trees. The SARS-CoV-2 genome OK244698 is similar, with the collection date changed from 14 January 2020 to 30 December 2021. Because it did not appear in two NCBI trees, the tree comparison method mentioned above would not detect the alteration of the collection date. Thus, keep in mind that the list of SARS-CoV-2 genomes with altered collection dates reported in this manuscript represents only a small fraction of those in GenBank with altered collection dates.

## 3. Results

### 3.1. Inauthentic SARS-CoV-2 Genomes in GenBank

While most SARS-CoV-2 genomes identical to the reference genome (NC_045512) were sampled in early 2020, at least nine such SARS-CoV-2 genomes were collected from 2021 to 2024 (Table 1). They are all exact copies of NC_045512, with a sequence length of 29,903. As shown in the last column of Table 1, the probability for such occurrences is effectively 0.

One can appreciate such probability statements intuitively. The reference genome NC_045512 belongs to the CCCA lineage (where CCCA stands for the four nucleotides at sites 241, 3037, 14,408, and 23,403, respectively, following the numbering of the reference genome NC_045512) [31,32]. This lineage was rapidly replaced by the D614G lineage, characterized by TTTG at the four sites mentioned above [31]. There were 1262 SARS-CoV-2 genomes of length 29,903 sampled between 1 April 2023 and 31 January 2024. The chance of the exact original NC_045512 being sampled in 2021–2024 and sequenced with 0 error, even just once, is extremely small, let alone multiple times, as shown in Table 1. The five most unlikely genome sequences were from India (Table 1). These five genomes, with a sampling time ranging from 10 April 2023 to 18 January 2024, are exact copies of NC_045512.

One genome from the USA (OM094978) was sampled on 24 March 2021. The USA contributed a total of 863 genomes of length 29,903 to GenBank in March 2021. Thus, the chance of obtaining a genome such as OM094978 is effectively 0. Similarly, the USA contributed four genomes of length 29,903 to GenBank in October 2021, which also implies an extremely small probability of obtaining a genome such as OP022337 that is an exact copy of the reference genome NC_045512 (Table 2).

There are also multiple SARS-CoV-2 genomes identical to NC_045512 that were sampled in late 2020 (Table 2). A total of 98,218 SARS-CoV-2 genomes of length 29,903 were sampled between 1 October 2020 and 30 December 2020. Thus, the chance of obtaining even just one sequence identical to NC_045512 in November and December of 2020 is very small, let alone the 13 sequences shown in Table 2. Two genomes sampled in December 2020 were from Pakistan (Table 2), out of eight genomes of length 29,903 submitted from Pakistan in December 2020. The USA contributed 13 genomes identical to NC_045512 (Table 2). From 1 October to 30 December 2020, the USA contributed 1887 SARS-CoV-2 genomes of length 29,903 to GenBank, which is far from sufficient to explain the 13 genomes identical to NC_045512. Thus, even a technologically advanced country could contribute SARS-CoV-2 genome sequences that are unlikely to be authentic.

The presence of those inauthentic genomes shown in Table 1 could dramatically affect the dating of the common ancestors of sequenced SARS-CoV-2 genomes and the estimation of the evolutionary rate. For example, the genome PQ008635 sampled on 18 January 2024 (Table 1) implies an extremely slow rate of evolution, without any nucleotide substitution or indels over more than four years. The inclusion of such sequences would lead to highly biased estimates of the evolutionary rate and the origin of the common ancestors of SARS-CoV-2.

### 3.2. Changes in Viral Sample Collection Times

Many changes in the collection dates were minor, with the date discrepancy smaller than five days. I list those date changes for SARS-CoV-2 genomes with date discrepancies equal to or greater than five days in Table 3. Most of the changes in the collection dates were due to the wrong entry of the year, i.e., 2021 entered as 2020 (Table 3). Of the two genomes submitted by Iranian scientists, the discrepancy in the original and the modified dates was attributed to the usage of different calendars. I should mention that many changes in collection dates may not be revealed by the comparison of collection dates between NCBI-generated phylogenetic trees, as described in Section 2.

For the first three genomes in Table 3, I happened to have downloaded their GenBank files twice, and they differed in collection times. The alterations of the sample collection times for the rest of the genomes in Table 3 were detected from the comparison of NCBI trees that I explained in Section 2.

The first 26 genomes in Table 3 are all typical D614G strains, with TTTG present at sites 241, 3037, 14,408, and 23,403, respectively, following the numbering of the reference genome NC_045512. The original wrong dates in these genomes would lead one to infer that the D614G strains occurred quite early, almost simultaneously circulating with the CCCA strain. Had one included these genomes with the original wrong dates in tip-dating, one would tend to date the common ancestor to a date earlier than it should.

Sometimes, the submitter would want to replace a submitted SARS-CoV-2 genome with another genome, e.g., replacing MT276328.2 with MT304487. The two may have different sample collection times, e.g., MT276328.2 with a collection time of 27 February 2020 replaced with MT304487 with a collection time of 1 March 2020. This could happen when the genome was re-sequenced. GenBank does not keep a record of such changes, nor does it ask for reasons for change. This causes not only confusion but also discrepancies in the results of genomic sequence analysis.

### 3.3. NCBI Is Slow to Correct Annotation Errors

I will use SARS-CoV-2 genomes derived from minks to illustrate the slowness in correcting errors in genomic sequence annotation. There are many mink-derived SARS-CoV-2 genomes [33]. In many of these mink-derived genomes (e.g., MT457390 to MT457401), the host was annotated as *Mustela lutreola* (European mink). However, all SARS-CoV-2 genomes from minks were from mink farms, and all farmed minks are American mink (*Neovison vison*). Thus, the annotation of the host species as *Mustela lutreola* is wrong. I contacted one of the submitters on 7 September 2021, and the submitter replied that they would correct the error. I waited until today (20 August 2024) and the error remains uncorrected. NCBI needs to have more resources to address the data curation problem.

The early reports on GenBank [34,35,36] emphasized the quality control and annotation of the submitted sequences. However, later reports [37,38,39] hardly mentioned quality control and annotation but instead highlighted how many billion sequences and how many trillion nucleotides were deposited in the database. While the exponentially increasing number of sequences and nucleotides stored in GenBank is a good thing, sometimes quality cannot be compensated by quantity in science.

## 4. Discussion

This is the first paper that casts doubt on the authenticity of genome sequences submitted to GenBank. I originally suspected that those sequences in Table 1 were likely from frozen meat, i.e., an original SARS-CoV-2 virus in Wuhan was frozen in its evolution but was isolated more than four years later by food inspectors. Unfortunately, this was not true. For example, the last five SARS-CoV-2 genomes in Table 1 were isolated from stool, saliva, nasopharyngeal, sewage, and stool, respectively. The hosts were all annotated as human, although it is unclear how a viral sample isolated from sewage could be ascertained to have a human source. It would take multiple miracles for them to be identical to the reference genome NC_045512 after four years, i.e., they cannot possibly be authentic. One cannot help asking how many sequences in GenBank are not authentic, considering that even a low-power analysis can detect so many impossible sequences. Can we still trust GenBank? NCBI needs to find more human resources to implement stringent quality control, otherwise there will be many incorrect conclusions in publications.

Bioinformatic analysis of sequences typically would take sequencing errors into consideration and suggest quality control measures [18,19,20,21,22]. However, it would be too much to ask bioinformaticians to consider the possibility that the sequences may not be authentic when it is nearly impossible to validate the authenticity of the submitted sequences. For example, if the SARS-CoV-2 genomic sequences in Table 1 and Table 2 were not exact copies of the reference genome NC_045512, or if they were submitted to GenBank in 2020 instead of 2024, then one would have neither a biological nor statistical foundation to claim that they are unlikely authentic.

Statistical inference and bioinformatic analysis depend heavily on the quality of data. As I have shown, there are errors and uncertainties in the submitted SARS-CoV-2 genomes. Uncertainty in genome annotation can dramatically affect our conclusions. For example, two SARS-CoV-2 genomes from Japan (MW219695 and BS001049) have the same collection time of 1 February 2020 (as of today, 20 August 2024), but MW219695 belongs to the CCCA clade and BS001049 to the TTTG/D614G clade. The two differ by 28 nucleotides. If the collection dates are correct, then we can infer that the TTTG/D614G lineage must have been co-circulating with the Wuhan CCCA lineage simultaneously around the time of the Wuhan outbreak. This would suggest that most published papers on SARS-CoV-2 evolution are incorrect. However, if we cannot be certain of the collection date, it is possible that BS001049 actually had a later collection date but with an incorrectly entered collection date of 1 February 2020. The conventional wisdom in the early phase of the COVID-19 pandemic is that the TTTG/D614G lineage is a late derivative, descending from the early CCCA lineage that caused the Wuhan outbreak [25,40,41]. In this framework, the TTTG/D614G genomes, such as BS001049, with an early collection time are typically assumed to have a wrong collection time (i.e., the true collection time was sometime later than the reported date). However, the TTTG/D614G lineage and the intermediate forms between the CCCA lineage and the TTTG/D614G lineage were subsequently isolated in China and Germany as early as January 2020 [32,42], and increasing evidence favors the hypothesis of the CCCA and TTTG/D614G lineages co-circulating before the Wuhan outbreak [43]. All these uncertainties would disappear if SARS-CoV-2 genomes in GenBank had accurate sample collection times. It is time for NCBI to demand all submitters of sequences to provide a detailed description of the submitted data, as has been previously suggested [44]. An incentive for this is to render the data description citable.

One might wonder if alternative databases, such as the viral database GISAID [45,46], might have better quality control to filter out inauthentic sequences, such as those in Table 1. Unfortunately, this is not true. For example, the last four genomes in Table 1 (PQ008636, PQ008633, PQ008634, and PQ008635) can also be found in GISAID, with their GISAID IDs being EPI_ISL_19262563, EPI_ISL_19262561, EPI_ISL_19262564, and EPI_ISL_19262562, respectively. GISAID currently has 17 million SARS-CoV-2 genomes. Detecting one or a few outliers is easier when all other sequences are high-quality authentic ones than when many inauthentic sequences are deposited, which would blur the difference between the authentic and inauthentic sequences.

## 5. Conclusions

This paper revealed many errors in both sequences and sequence annotations in SARS-CoV-2 genomes submitted to GenBank. Because the method could only detect a small fraction of errors in sequences and sequence annotations, the real number of errors could be much greater than revealed in this paper. There is an urgency for NCBI to implement stringent quality control in genome submissions, especially when public health depends on the quality of such sequences and sequence annotations.

## Figures and Tables

**Table 1:** At least nine SARS-CoV-2 genomes collected during 2021–2024 and deposited in GenBank were identical to the reference genome, NC_045512, and unlikely to be authentic.

ACCN ^(1)^	Country	T ^(2)^	δ ^(3)^	λ ^(4)^	$f\left(0|\lambda \right)$ ^(5)^
OM094978	USA	24 March 2021	454	25.0880	1.2718 × 10^−11^
OM108445	India	1 July 2021	553	30.5588	5.3517 × 10^−14^
OP022337	USA	20 October 2021	664	36.6926	1.1603 × 10^−16^
OP268178	Mexico	19 August 2022	967	53.4364	6.2067 × 10^−24^
PP434597	India	10 April 2023	1201	66.3673	1.5034 × 10^−29^
PQ008636	India	10 December 2023	1445	79.8507	2.0955 × 10^−35^
PQ008633	India	11 January 2024	1477	81.6190	3.5753 × 10^−36^
PQ008634	India	11 January 2024	1477	81.6190	3.5753 × 10^−36^
PQ008635	India	18 January 2024	1484	82.0058	2.4284 × 10^−36^

(1) GenBank accession number. (2) Sample collection time. (3) Time interval in days between 26 December 2019 (collection time for NC_045512) and time T. (4) Expected number of nucleotide replacements during the period *δ*. (5) The probability that the genome identical to NC_045512 was sampled at the collection time, based on the calculation in Equations (1) and (2).

**Table 2:** Fifteen SARS-CoV-2 genomes sampled in late 2020 and deposited in GenBank were identical to the reference genome NC_045512. Column headings are the same as in Table 1.

ACCN	Country	T	δ	λ	f(0|λ)
OM095202	USA	8 October 2020	287	15.8596	1.2950 × 10^−07^
MZ722043	USA	25 October 2020	304	16.7990	5.0614 × 10^−08^
OM095001	USA	25 November 2020	335	18.5121	9.1264 × 10^−09^
OM095004	USA	25 November 2020	335	18.5121	9.1264 × 10^−09^
OM095010	USA	25 November 2020	335	18.5121	9.1264 × 10^−09^
OM095127	USA	11 December 2020	351	19.3963	3.7697 × 10^−09^
MW960278	Pakistan	11 December 2020	351	19.3963	3.7697 × 10^−09^
MZ722192	USA	14 December 2020	354	19.5620	3.1938 × 10^−09^
OP278726	Pakistan	17 December 2020	357	19.7278	2.7059 × 10^−09^
OM095142	USA	21 December 2020	361	19.9489	2.1693 × 10^−09^
MZ722000	USA	21 December 2020	361	19.9489	2.1693 × 10^−09^
MZ722615	USA	21 December 2020	361	19.9489	2.1693 × 10^−09^
MZ722630	USA	21 December 2020	361	19.9489	2.1693 × 10^−09^
MZ722702	USA	21 December 2020	361	19.9489	2.1693 × 10^−09^
OP022336	USA	30 December 2020	370	20.4462	1.3193 × 10^−09^

**Table 3:** A partial list of SARS-CoV-2 genomes deposited in GenBank with modified collection times that differ from the original by ≥5 days.

ACCN	Country	T1 ^(1)^	T2 ^(2)^	Tree1..Tree2 ^(3)^	T1–T2
MW795884	USA	13 January 2020	13 January 2021		−366
OK244698	USA	14 January 2020	30 December 2021		−716
MW585340	USA	5 January 2020	5 January 2021		−366
MZ028629	USA	18 February 2020	18 February 2021	12 July 2021..7 May 2022	−366
MZ436887	Sierra Leone	14 January 2020	14 January 2021	8 November 2021..7 May 2022	−366
MZ436896	Sierra Leone	14 January 2020	14 January 2021	8 November 2021..7 May 2022	−366
MZ469886	USA	12 January 2020	12 January 2021	8 November 2021..7 May 2022	−366
MZ469887	USA	6 January 2020	6 January 2021	8 November 2021..7 May 2022	−366
MZ473469	USA	17 February 2020	17 February 2021	8 November 2021..7 May 2022	−366
MW786995	USA	10 March 2020	10 March 2021	3 April 2021..7 May 2022	−365
MW921831	USA	15 March 2020	15 March 2021	25 April 2021..7 May 2022	−365
MZ021503	India	1 March 2020	1 March 2021	8 November 2021..7 May 2022	−365
MZ021504	India	6 March 2020	6 March 2021	8 November 2021..7 May 2022	−365
MZ021505	India	6 March 2020	6 March 2021	8 November 2021..7 May 2022	−365
MZ021506	India	6 March 2020	6 March 2021	8 November 2021..7 May 2022	−365
MZ278198	USA	21 April 2020	21 April 2021	8 November 2021..7 May 2022	−365
MZ397171	Myanmar	28 May 2020	28 May 2021	8 November 2021..7 May 2022	−365
MZ397172	Myanmar	28 May 2020	28 May 2021	8 November 2021..7 May 2022	−365
MZ397173	Myanmar	28 May 2020	28 May 2021	8 November 2021..7 May 2022	−365
MZ397174	Myanmar	28 May 2020	28 May 2021	8 November 2021..7 May 2022	−365
MZ397175	Myanmar	2 June 2020	2 June 2021	8 November 2021..7 May 2022	−365
MZ397176	Myanmar	2 June 2020	2 June 2021	8 November 2021..7 May 2022	−365
MZ397177	Myanmar	26 May 2020	26 May 2021	8 November 2021..7 May 2022	−365
MW591579	USA	18 January 2020	17 December 2020	25 April 2021..7 May 2022	−334
MW750862	USA	22 May 2020	2 March 2021	3 April 2021..7 May 2022	−284
MW750906	USA	23 May 2020	14 January 2021	3 April 2021..7 May 2022	−236
MW737421	Iran	25 October 2019	11 January 2020	3 April 2021..7 May 2022	−109
MW898809	Iran	12 December 2019	29 February 2020	25 April 2021..7 May 2022	−79
MZ077094	USA	14 April 2021	20 April 2021	12 July 2021..7 May 2022	−6
MW093534	USA	6 June 2020	11 June 2020	3 April 2021..4 September 2021	−5
MW883366	USA	29 March 2021	22 March 2021	25 April 2021..7 May 2022	7
MW883371	USA	27 March 2021	16 March 2021	25 April 2021..7 May 2022	11
MW883363	USA	29 March 2021	11 March 2021	25 April 2021..7 May 2022	18
MW883370	USA	27 March 2021	8 March 2021	25 April 2021..7 May 2022	19
MW883364	USA	29 March 2021	21 January 2021	25 April 2021..7 May 2022	67

(1) Sample collection dates recorded in an earlier tree. (2) Sample collection dates in a later tree. (3) Two trees downloaded at two dates, shown in the form of “Date1..Date2”. The first three genomes were from my communication with the submitters of the GenBank genomes (i.e., not from the comparisons of collection times of genomes between NCBI-generated trees).

## Data Availability

Data are contained within the article.
